# Protocol for the development and validation of a patient reported measure (PRM) of treatment burden in stroke

**DOI:** 10.12688/healthopenres.13334.1

**Published:** 2023-06-02

**Authors:** Katie I Gallacher, Martin Taylor-Rowan, David T Eton, Hamish McLeod, Lisa Kidd, Karen Wood, Aleema Sardar, Terry J Quinn, Frances S Mair

**Affiliations:** 1 General Practice and Primary Care, University of Glasgow, Glasgow, Scotland, G12 8TB, UK; 2 Outcomes Research Branch, Healthcare Delivery Research Program, Division of Cancer Control and Population Sciences, National Cancer Institute, Bethesda, USA; 3 Mental Health and Wellbeing, Gartnavel Royal Hospital, Glasgow, Scotland, G12 0XH, UK; 4 Department of Nursing and Community Health, Glasgow Caledonian University, Glasgow, Scotland, G40BA, UK; 5 School of Medicine, University of Glasgow, Glasgow, Scotland, UK; 6 Institute of Cardiovascular and Metabolic sciences, University of Glasgow, Glasgow, Scotland, UK

**Keywords:** Stroke, Treatment Burden, Validity, PETS-stroke

## Abstract

**Background:**

Treatment burden is the workload of healthcare for people with long-term conditions and the impact on wellbeing. A validated measure of treatment burden after stroke is needed. We aim to adapt a patient-reported measure (PRM) of treatment burden in multimorbidity, PETS (Patient Experience with Treatment and Self-Management version 2.0), to create a stroke-specific measure, PETS-stroke. We aim to examine content validity, construct validity, reliability and feasibility in a stroke survivor population.

**Methods:**

1) Adaptation of 60-item PETS to PETS-stroke using a taxonomy of treatment burden. 2) Content validity testing through cognitive interviews that will explore the importance, relevance and clarity of each item. 3) Evaluation of scale psychometric properties through analysis of data from stroke survivors recruited via postal survey (n=340). Factor structure will be tested with confirmatory factor analysis and Cronbach’s alpha will be used to index internal consistency. Construct validity will be tested against: The Stroke Southampton Self-Management Questionnaire; The Satisfaction with Stroke Care Measure; and The Shortened Stroke Impact Scale. We will explore known-groups validity by exploring the association between treatment burden, socioeconomic deprivation and multimorbidity. Test-retest reliability will be examined via re-administration after 2 weeks. Acceptability and feasibility of use will be explored via missing data rates and telephone interviews with 30 participants.

**Conclusions:**

We aim to create a validated PRM of treatment burden after stroke. PETS-stroke is designed for use as an outcome measure in clinical trials of stroke treatments and complex interventions to ascertain if treatments are workable for patients in the context of their everyday lives.

## Introduction

The term *treatment burden* describes the personal healthcare workload of living with long-term conditions and the impact of this work on wellbeing, usual roles, and daily activities. Treatments can become burdensome when there are too many, or if it is difficult to implement them in everyday life. People with stroke describe the workload of healthcare as pervasive and draining of time, energy and finances ^1^. In addition to the arduous work required during stroke rehabilitation and the lifelong therapies that follow ^1^, stroke survivors may have physical, cognitive, or emotional impairments that increase the work of health self-management ^2^. There has been a recent interest in understanding treatment burden and developing methods of measurement, to aid identification of high-risk groups and assist the testing of interventions. 

In previous research we created a conceptual model and taxonomy of treatment burden from the stroke survivor perspective ^2^. Stroke survivors reported four potentially burdensome categories of healthcare work: sense making, interacting with others, enacting treatments and reflecting on progress ^1^. An important finding was that treatment burden is often iatrogenic, resulting from either an increased healthcare workload imposed by healthcare providers e.g. multiple healthcare appointments, or deficiencies in the way care is provided e.g. poor communication between health professionals ^2^. Treatment burden after stroke is influenced by a person’s ability to manage their health, which is affected by: personal skills and attributes; physical and cognitive abilities; social support; financial status; life workload; and environment ^2, 3^.

Treatment burden is a subjective phenomenon therefore patient-reported methods are suitable for measurement. A recent systematic review identified no comprehensive patient-reported measure (PRM) of treatment burden in stroke ^4^, however PRMs of treatment burden in multimorbid populations with no index condition have been developed ^5^. These have proven useful in identifying the generic treatment burdens associated with long-term condition management but omit important stroke-specific burdens. One such PRM is the Patient Experience with Treatment and Self-Management (PETS), developed to measure treatment burden in people with multimorbidity and/or complex self-management regimens ^5^. The PETS was rigorously developed and tested, and its content aligns with our own conceptual model of treatment burden. However, as it omits stroke-specific burdens, amendment and validation in a European stroke survivor population is required. Stroke survivors tend to be older, frailer, less affluent and more cognitively impaired than participants in the PETS validation study population. There are also important differences between European and US healthcare systems that may influence the experience of treatment burden. The first-generation version of the PETS had 46 items (Eton *et al.*, 2017) and the next generation version, the PETS 2.0, has 60-items ^6^. We will adapt PETS 2.0 to create a PRM of treatment burden for use in stroke survivors – the PETS-Stroke.

## Aim

The aim of this project is to adapt, refine and validate the PETS measure to develop a new PRM of treatment burden for use with stroke survivors. We will answer the following research questions:

1) What items should be included in PETS-stroke?

2) What is the validity of PETS-stroke?

3) How reliable is PETS-stroke?

4) What factors affect feasibility of using PETS-stroke?

## Methods

**Figure 1.**
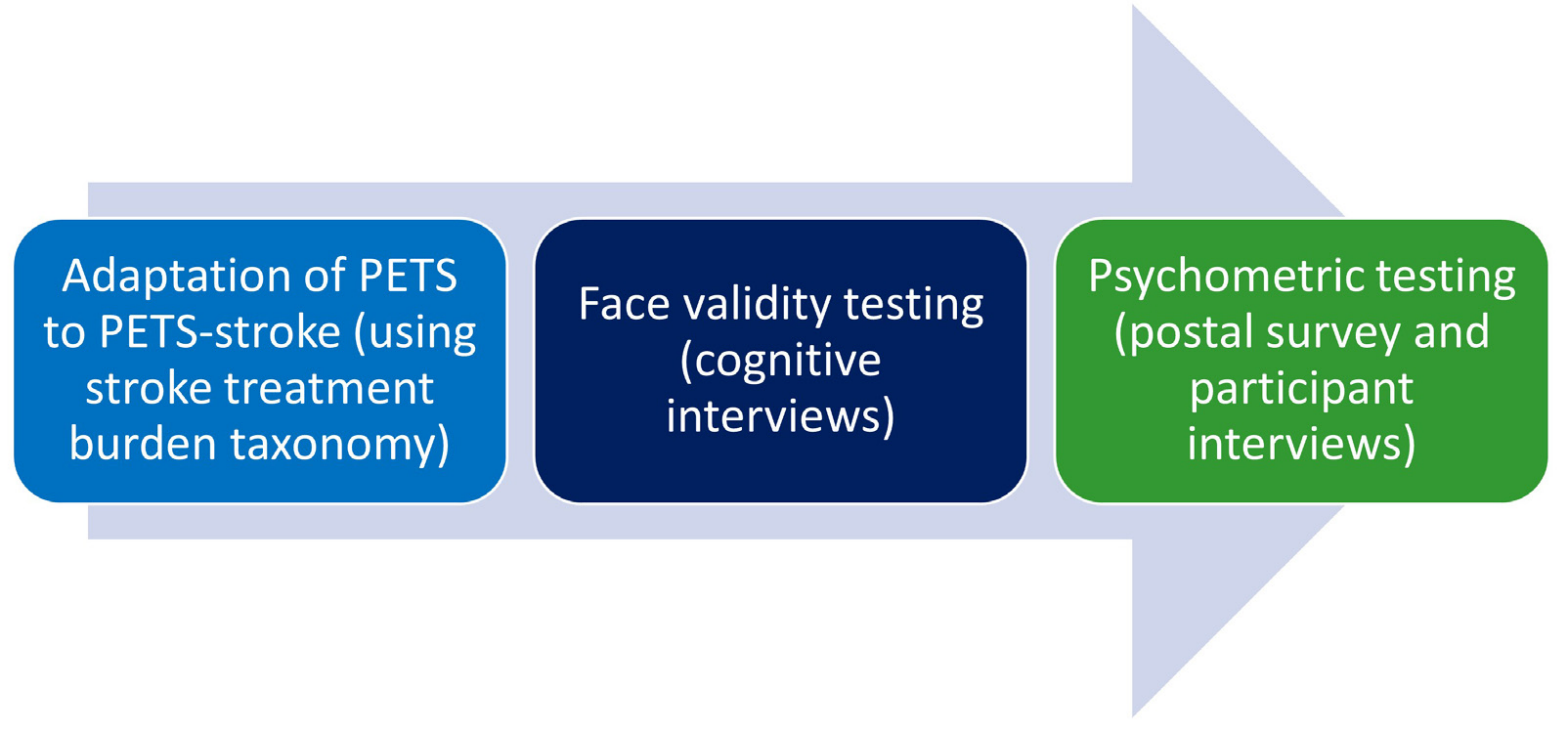
Summary of PET-stroke development & validation process.

Ethical approval has been provided on 16/9/20 by London and Surrey Borders NHS Research Ethics Committee (20/LO/0871). Guidance on PRM development published by the International Society for Quality of Life Research (ISOQOL) ^7^ and COSMIN ^8^ have informed methodology. A summary of each stage of the project can be seen in Figure 1.

### Stage 1 - Adaptation of the PETS

**Figure 2.**
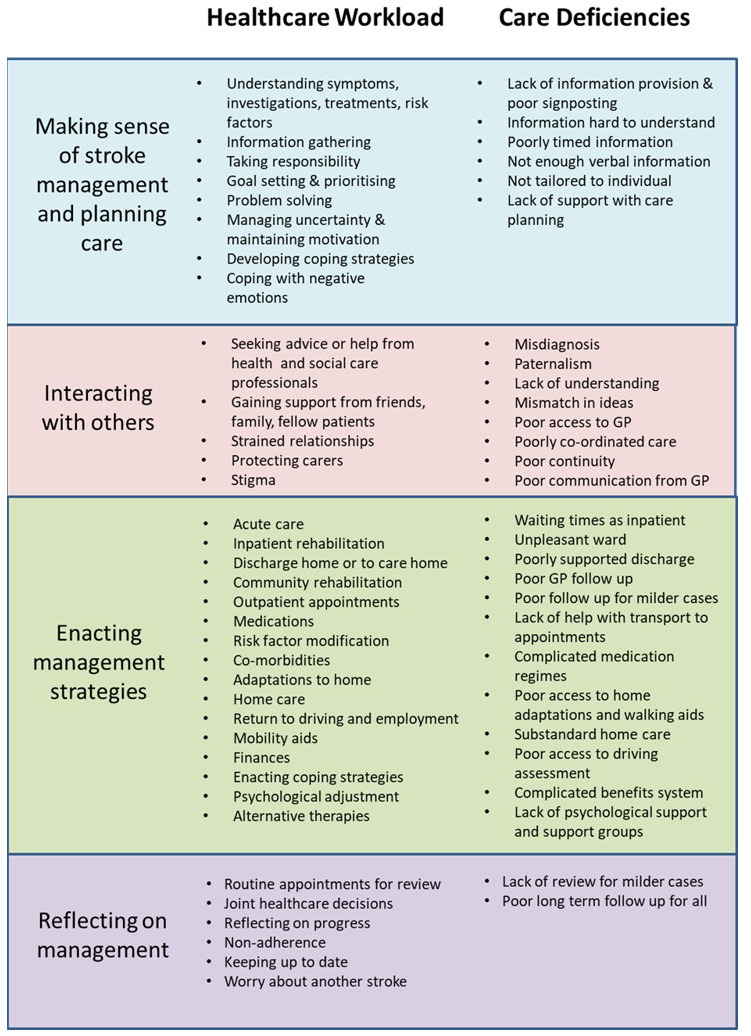
Taxonomy of treatment burden in stroke. Reproduced from Gallacher, K.I., May, C.R., Langhorne, P. *et al*. A conceptual model of treatment burden and patient capacity in stroke. BMC Fam Pract 19, 9 (2018). https://doi.org/10.1186/s12875-017-0691-4 reproduced under Creative Commons Attribution 4.0 International License ( http://creativecommons.org/licenses/by/4.0/).

**Figure 3.**
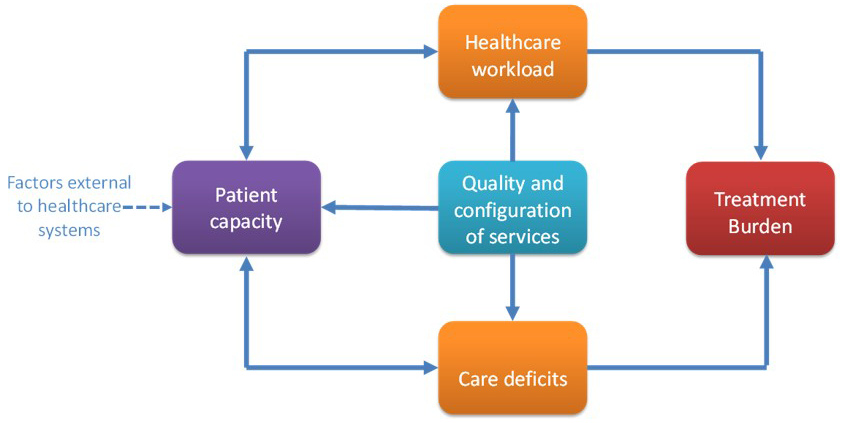
Conceptual model of treatment burden in stroke. Reproduced from Gallacher, K.I., May, C.R., Langhorne, P. et al. A conceptual model of treatment burden and patient capacity in stroke. BMC Fam Pract 19, 9 (2018). https://doi.org/10.1186/s12875-017-0691-4 under Creative Commons Attribution 4.0 International License ( http://creativecommons.org/licenses/by/4.0/).

Item generation will be conducted by adapting items in PETS 2.0 using our conceptual model and taxonomy of treatment burden created from our previous qualitative work ^1, 9^ ( Figure 2, Figure 3). This will be done through discussion between members of the research team which includes international experts in treatment burden and clinicians involved in stroke care. PETS 2.0 contains 60 items with 14 individual domains consisting of two individual-item domains and 12 scales containing two to seven items each. To limit assessment burden on respondents we aim to reduce the number of total items whilst also adapting to ensure stroke-relevant content. The planned output from this stage is a prototype of the new PETS-Stroke.

### Stage 2 - Content validity testing

Cognitive interviews will be undertaken with approximately 10-20 stroke survivors to ascertain if the content of each item in PETS-stroke is important, relevant, and coherent, and if any treatment burdens are omitted. Inclusion criteria: diagnosis of stroke; being able to read and communicate in English; and being able to provide informed consent. People with aphasia or mild cognitive difficulties will be included. Participants will have the option of being supported by a proxy. Those with a prognosis under six months will be excluded. Purposive sampling will be conducted, aiming to recruit a varied group of stroke survivors according to sex, age, time since stroke and level of disability/aphasia.

For content validity testing, participants will be recruited from local stroke groups and from NHS primary care practices in Greater Glasgow and Clyde through the NRS Primary Care Research Network. 

Cognitive interviews will involve the researcher and participant collaboratively reading through the content of each item to establish if it is fit for purpose ^10, 11^. Interviews will be conducted either in person or through video or telephone call. Written informed consent will be taken at the start of the interview. Interviews will be recorded and transcribed verbatim, then analysed by framework analysis to explore importance, relevance, clarity and recall period of each item. Data will be analysed after five interviews as this is deemed a useful yet manageable amount of data, then amendments made to the PRM-based on data analysis, and the amended version shown to the subsequent five participants with further changes made based on those interviews. Interviews will continue in this manner until data saturation is achieved ^12^ and no new themes identified. The output from this stage will be a version of PETS-stroke that has been shown to be important, relevant, and coherent to a group of stroke survivors.

### Stage 3 – Construct validity and reliability testing

This stage will involve asking a large group of stroke survivors to complete the PETS-stroke measure, presented as a self-administered, paper-and-pencil survey.

***Recruitment.*** We will recruit stroke and transient ischaemic attack (TIA) survivors, using the diagnosis made by the individual’s clinical team. The decision to include both stroke and TIA survivors for this stage is due to the clinical challenges that can be faced in distinguishing TIA and mild stroke, the resultant challenge of accurate coding in medical records, the similar treatments for both conditions, and the lack of face-to-face contact with the research team which makes clarification difficult. We will ensure participants with a wide range of post-stroke disabilities are included to avoid a sample with over representation of TIAs or mild strokes. Inclusion criteria: community dwelling (including retirement housing or sheltered housing); stroke diagnosis (including ischaemic, haemorrhagic, any severity and TIA); over one month since hospital discharge at time of completing the survey (in order to capture treatment burdens encountered at home rather than in the hospital, those not admitted to hospital will also be included); able to read English; able to complete the paper survey or ask someone to do as a proxy. Exclusion criteria: subarachnoid haemorrhage; severe aphasia; cognitive difficulties severe enough to preclude an interview; not being able to give informed consent; at end of life. Findings from the cognitive interviews in stage 2 will further inform inclusion and exclusion criteria, for example time since stroke or stroke severity may affect relevance of the PRM. A sampling frame will be utilised to promote diversity in participant characteristics (age, sex, ethnicity, socioeconomic status, stroke sequalae, time since stroke) and generalisability of the sample will be examined at 25% and 75% recruitment using data from the Scottish Stroke Care Audit as a comparator ^13^. Proactive steps will be taken to enhance recruitment of any underrepresented groups. 

Recruitment through the NHS will be conducted through hospital wards, stroke clinics, community stroke teams and stroke registries at ten participating hospitals in Scotland and one in Wales. Two sites have been added subsequently to the others with appropriate ethical amendments sought and approved ( Table 1). Similarly, recruitment through community stroke teams and stroke registries was a later addition. We initially planned to recruit for 14 months but this has been increased to 17 months after discussion with sites to achieve our planned sample size. In Scotland, the NRS Stroke Research Network will conduct the majority of recruitment with some sites also utilising clinical staff. In Wales recruitment will be conducted by Cardiff and Vale University Health Board staff. Additional recruitment will be conducted through the Scottish Health Research Register (SHARE) ^14^, and via advertisements on social media (Twitter). SHARE maintain a register of people interested in participating in health research who have agreed to allow their coded data in NHS records to be utilised to check if they are suitable for research projects. SHARE will identify suitable individuals using our inclusion criteria and contact them to ask if they are willing to take part. Details of those who are agreeable will be sent to the research team who will then send out a survey pack. This multi-pronged recruitment approach will help to improve sample adequacy and recruitment of traditionally underrepresented population cohorts (e.g. people with low literacy and/or from economically deprived regions).

**Table 1.** Participating hospital sites.

Glasgow Royal Infirmary
Queen Elizabeth University Hospital
University Hospital Monklands
University Hospital Hairmyres
Forth Valley Royal Hospital
Aberdeen Royal Infirmary
Victoria Hospital
Ninewells Hospital
Royal Alexandra Hospital *
University Hospital of Wales *

*Added as sites in amendment

Survey packs will be administered in person or posted out to potentially eligible participants. When administering the packs in person, consent will be gained to contact the person 2–4 weeks later to check understanding. Participants who are identified through local registry data will be invited by letter followed by a telephone call. For those recruited through SHARE, the third sector and social media, packs will be posted out. Potential participants will have access to the research team by telephone for any questions. Participants will be able to change their mind and withdraw at any point in the process. A £10 gift voucher will be offered to respondents for return of completed questionnaires ^15^.

If the survey is sent back too early (within 4 weeks) after hospital discharge or has a lot of missing data, the participant will be invited to complete another survey.

***Consent.*** Those recruited through the NRS Stroke Research Network will have the option of consent being gained prior to discharge from hospital. Those not consented at that stage and those recruited through SHARE or social media will complete the consent form when completing the survey.

***Data collection.*** Stroke survivors will be asked to complete the survey packs at home and return to the research team in a prepaid envelope. Packs will include the PETS-stroke measure along with three additional PRMs: The Stroke Southampton Self-Management Questionnaire ^16^; The Satisfaction with Stroke Care Measure ^17^; and The Shortened Stroke Impact Scale ^18^. In addition, self-reported demographic data will be requested including: date of birth, gender, ethnicity, level of educational attainment, post code, date of stroke, date of discharge from hospital (if admitted), history of medical conditions, ongoing medical prescriptions, participation in other ongoing research studies or trials, ongoing functional issues, whether they live alone, and whether they have support with tasks or chores when necessary.

It will be emphasised that a carer can support the person completing the survey if they are acting as a proxy for the person rather than reporting their own viewpoint. This will enable those with mild aphasia or physical disabilities that make writing difficult to still take part. Data will be collected on whether the participant has any assistance in completing the survey. For test-retest reliability, we will send our PRM out a second time 2 weeks after return of the first questionnaire.

***Sample size.*** To guide our decision about recruitment target we assume factor loadings of 0.5 and factor correlations of 0.3 and an average of 3 indicators per factor. Using Monte Carlo simulations, Wolf *et al*. ^19^ present sample sizes for between 1 and 3 factors. Our projected sample size of 340 participants gives more than adequate power to test the two factor (workload and impact) superordinate model reported on the PETS 2.0 (Lee *et al*., 2020). Based on the simulations of Wolf *et al.* ^19^ we will also have sufficient sample to fit a 12 factor model derived from the PETS 2.0. Wolf *et al.* demonstrate that there is little or no change in power going from 2 to 3 factors (in contrast to a large change from 1 to 2 factors). So, our projected sample size of 340 participants gives a ratio of approximately 10 participants per item and provides a comparable sample to the CFA study of the PETS 2.0 ^6^.

We will monitor both the return rates for the baseline questionnaires and the rate of item completion to ensure that we obtain 340 questionnaires with <10% incomplete data on each item and recruit further if needed. Furthermore, we have carried out a sample size calculation for reliability to assess the potential impact of attrition at follow-up: to estimate a 95% confidence interval of width 0.2 (i.e. 0.4-0.6) around a conservative intraclass correlation coefficient (ICC) of 0.5, we would require 218 participants to provide questionnaires at both baseline and follow-up, equating to 64% of the overall sample, and we are confident we can achieve this. In addition, we anticipate an ICC of higher than 0.5, which would require fewer completed questionnaires for the same width confidence interval. 

***Data analysis.*** PETS 2.0 has 60 items (14 domains) with 12 first-order factors representing individual content domains and two second-order factors representing superordinate factors of highly-correlated individual domains (Workload and Impact) (Lee *et al*., 2020). Two single indicator items were set aside for the factor analysis (Monitoring Health and Mental Fatigue). The main analyses will focus on testing the factor structure of the PETS-Stroke starting with the bifactor and multiple factor models reported by Lee *et al.*, 2020 ^6^ providing the basis for model specification. We will also examine the psychometric properties of the scale including subscale internal consistency, test-retest stability, and construct validity (via comparison with existing scales that assess aspects of post-stroke burden and distress). All analyses and assumptions will be described in a detailed *a priori* publicly available Statistical Analysis Plan.

***Confirmatory factor analysis.*** The model parameters for the CFA will be specified based on the factor structure of the PETS 2.0 and agreed by the core study team (which is comprised of experts in stroke healthcare). We will proceed to EFA using the collected sample if we fail to observe an identified and well-fitting model with CFA. Before analyses, patterns of missing data will be scrutinised by the study team and examined with Little’s MCAR. Where appropriate, imputation methods will be used to address missing data and to minimise list-wise deletion. All data will be prepared by screening for univariate and multivariate normality and the presence of outliers. As the observed variables are measured on ordinal scales, we will conduct the analysis on polychoric correlation matrices and weighted least squares with adjustments for mean and variance (WLSMV). Model fit evaluation will be determined by multiple indices (model χ ^2^, WRMR≥1.0, RMSEA≥0.08, TLI/CFI≥0.95) in line with best practice guidance ^20, 21^ and we will compare the comparative fit of the bifactor and multifactor models using Akaike’s information criterion (AIC), Bayesian information criteria (BIC) and sample size adjusted BIC. Any post hoc model modifications or adjustments made to improve fit will be reported in full in the main outcomes paper. Analysis will be carried out in R using the packages Lavaan (v 0.6-13 or later) and semTools (v 0.5-6 or later).

Construct validity will be explored by correlating the PETS-Stroke second-order bifactor scores (Workload and Impact) to scores from the three other PRMs which are all valid and reliable measures in people with stroke. We hypothesize that increased levels of treatment burden will be correlated with lower readiness to self-manage, lower satisfaction with stroke services and increased burden of illness. Known-group validity will be assessed by looking at associations between socioeconomic status and treatment burden and additionally self-reported multimorbidity and treatment burden (our hypothesis being that those who are more deprived or multimorbid will have higher levels of treatment burden) ^7, 8, 22^.

Cronbach’s alpha will be used to examine internal consistency reliability of content domains ^7, 8, 22^. For test-retest reliability, we will calculate intraclass correlation coefficients (ICCs) between the first and second attempts at PRM completion for each participant ^7, 8, 22^.

To aid the interpretation of score meanings we will examine the distribution of responses and will use the scale anchors to propose thresholds for low and high scores that could be validated in future samples ^7, 8, 22^. Percentage of missing items and proportion of returned surveys will provide information on acceptability, and a subset of 30 participants will be interviewed by telephone after completion of the questionnaire to further explore this (they will tick a box on the form to opt in) ^22^.

### Stage 4 – Feasibility testing

Semi-structured interviews will be conducted by telephone with a subset of participants (approximately 30, guided by data saturation) ^12^ to enquire about usability of the survey. Examples of topics that will be discussed include time taken to complete the survey, wording of instructions and items, mode of survey (paper and pencil), and whether any breaks or help from a proxy were required. Participants will be given the opportunity to provide any feedback they deem important outside the interview guide. Interviews will be audio recorded and transcribed verbatim. Data will be analysed using a codebook thematic analysis approach to look for key themes or topics arising ^23^.

***Additional telephone interviews.*** If necessary, we will conduct 10 telephone interviews with individuals who completed the questionnaire pack to explore possible reasons for unfavourable psychometric results should this occur, for example if there are large amounts of missing data. 

A return slip will be added to the survey pack, to be returned if an individual does not wish to complete the survey but would consent to a short telephone interview. We aim to interview 10 individuals to explore reasons for not participating. These interviews will be analysed using codebook thematic analysis ^23^.

***Patient and public involvement.*** Our programme of work is overseen by a research advisory panel including stroke survivors and carers. This panel meets regularly, and members discuss their own experiences of treatment burden, research goals and priorities and provide feedback on applications. The advisory group will continue to direct this program of research over the span of this project. Additionally, this research was designed with input from 12 stroke survivors. These individuals were mostly contacted through the Voices Scotland program delivered by Chest Heart & Stroke Scotland (CHSS), and one was a personal contact of a colleague.

## Discussion

### Dissemination

Engagement events will be held with stroke survivors, carers, stroke triallists and those who provide and plan stroke health and social care services. The stroke survivors and caregivers on our research advisory group will be invited to plan these events and attend to help with dissemination of results as well as take part in discussions about the results and future research. A plain English version of the study results will be produced for dissemination through our engagement events, the third sector and social media.

Findings will be further disseminated with health professionals through professional forums such as UK Stroke Forum, the European Life After Stroke Forum, and the Health Services Research UK Network. Dissemination to academic colleagues will be achieved through presentations at national and international stroke-related conferences and publications in peer-reviewed journals. Policy makers will be reached through government advisory committees such as the Scottish National Advisory Committee for Stroke.

### Outputs and future work

An important output here is the adaptation and validation of PETS-stroke for use in identifying and evaluating extent of treatment burden in a stroke survivor population. PETS-stroke will be appropriate for use as an outcome measure in clinical trials of stroke treatments and complex interventions to ascertain if treatments are workable for patients in the context of their everyday lives and do not lead to additional or adverse experiences of treatment burden. It has the potential to be used as the primary outcome measure in much needed trials of interventions aimed at reducing burden and enhancing self-management but could also be an important secondary outcome measure in any trial looking at a change to usual practice in stroke care. PETS-stroke will also have value as a baseline case-mix adjuster, especially for trials looking at complex interventions. It also has potential to become part of a ‘core outcomes set’ for use in clinical trials. Additionally, the measure will allow examination of how treatment burden after stroke is associated with health-related outcomes such as quality-of-life and further stroke.

## Study status

The study is currently ongoing. Recruitment began in March 2022 and is anticipated to conclude in August 2023.

## Data Availability

No underlying data are associated with this article. Zenodo: Consent forms for TRUSTED study. https://doi.org/10.5281/zenodo.7890355 ^24^ Data are available under the terms of the Creative Commons Attribution 4.0 International license (CC-BY 4.0).
