# Supplementary material for: Protocol for the development and validation of a patient reported measure (PRM) of treatment burden in stroke
Source: Health Open Res. 2023 Oct 12;5:17. Originally published 2023 Jun 2. [Version 2] doi: 10.12688/healthopenres.13334.2 (PMC11064975; doi:10.12688/healthopenres.13334.2)
Supplement: Peer review history

# Reviewer report: Reviewer response for version 2

**DOI:** 10.21956/healthopenres.14506.r27565

**Published:** 2023-10-16

**Authors:** Adrià Arboix

**Affiliations:** 1 Universitat de Barcelona, Barcelona, Spain

In this revised manuscript, the authors changed it as the reviewer suggested. Now this manuscript is suitable for indexing.

Is the study design appropriate for the research question?

Yes

Is the rationale for, and objectives of, the study clearly described?

Yes

Are sufficient details of the methods provided to allow replication by others?

Yes

Are the datasets clearly presented in a useable and accessible format?

Yes

Reviewer Expertise:

cerebrovascular diseases; acute stroke; vascular cognitive impairment; lacunar stroke

I confirm that I have read this submission and believe that I have an appropriate level of expertise to confirm that it is of an acceptable scientific standard.

# Reviewer report: Reviewer response for version 1

**DOI:** 10.21956/healthopenres.14418.r27253

**Published:** 2023-07-14

**Authors:** Anne Marie Lunde Husebø

**Affiliations:** 1 Public Health, University of Stavanger, Stavanger, Rogaland, Norway; 2 Research Group of Nursing and Health Sciences, Research Department, Stavanger University Hospital, Stavanger, Rogaland, Norway

Thank you for the opportunity to review this interesting study protocol. This is a helpful article on an important topic, which will be relevant to an international audience, and in particular to researchers aiming to explore and further develop patient reported outcomes and measures. I enjoyed reading the manuscript and have some comments to support the development of the work.

**Abstract:** The abstract is well written, sufficiently structured, and stated in a clear manner.

**Key words:** I suggest you include the methodological approach as a key word.

**Introduction:** The introduction section appears as informative and well built up, introducing the reader to the topic in a convincing manner. However, a reference following the first sentence describing treatment burden would be helpful, and references may be added following the last sentence in the first paragraph, on developing methods to understand treatment burden.

**Aim: **The study has a clear purpose, and well framed research questions. I particularly found the last question intriguing as feasibility of a measurement may not always be included in such a study protocol.

**Methods: **I find using both quantitative and qualitative methods highly appropriate when developing new measurements. There is no mentioning of which reporting checklist the authors use. This may be included in this section.

 The methods section is very informative on sampling, data collection and outcomes, sample size calculations, data analysis. The recruitment strategy seems comprehensive and will probably secure the estimated number of participants. There is no mention of how the methods will secure an appropriate response scale and recall time of the PES-Stroke. Earlier, and ongoing research has found that patients struggle with capture the instructions regarding recall time of 4 weeks, and the “Don’t apply to me-response options” creates missing without any context to as why they are missing. I suggest these issues are included in both stage 3 and 4 and discussed further.

 The study has gained ethical approval and is ongoing do treatment burden appear during a treatment trajectory would be interesting to explore.

**Discussion: **The plan for dissemination includes all stakeholders and the activities described are traditional and well tested. The PETS 2.0. is a complex PRM within chronic or long-term illness and requires literacy skills among study participants. Thus, the plan for adapting it into a PETS Stroke measure as described in this protocol is necessary, and if performed as described, have high potential to become a relevant tool for capturing treatment burden in stroke.

**Figures: **The figures are informative. Even though the methods section is written in a clear manner, I would welcome a figure giving an overview of the study with it’s 4 stages including procedures and participants would be helpful to the readers functioning as a visual model of the 4 stages.

Is the study design appropriate for the research question?

Yes

Is the rationale for, and objectives of, the study clearly described?

Yes

Are sufficient details of the methods provided to allow replication by others?

Yes

Are the datasets clearly presented in a useable and accessible format?

Yes

Reviewer Expertise:

Treatment burden, non-communicable diseases, health services research, digital healthcare services, mixed- methods, RCT, qualitative research, systematic reviews.

I confirm that I have read this submission and believe that I have an appropriate level of expertise to confirm that it is of an acceptable scientific standard.

# Response

**Published:** 2023-08-22

**Authors:** Martin Taylor-Rowan

**Affiliations:** General Practice and Primary Care, University of Glasgow, Glasgoe, UK

1. Key words: I suggest you include the methodological approach as a key word.

**Thank you we have added the words survey methods, psychometric analysis, and qualitative methods.**

 2. Introduction: The introduction section appears as informative and well built up, introducing the reader to the topic in a convincing manner. However, a reference following the first sentence describing treatment burden would be helpful, and references may be added following the last sentence in the first paragraph, on developing methods to understand treatment burden. **Thank you, we have added references as suggested.**

 3. Methods: I find using both quantitative and qualitative methods highly appropriate when developing new measurements. There is no mentioning of which reporting checklist the authors use. This may be included in this section.

**Thank you, we have added the following sentence: The COSMIN reporting checklist will be utilised for reporting the study.**

 4. There is no mention of how the methods will secure an appropriate response scale and recall time of the PES-Stroke. Earlier, and ongoing research has found that patients struggle with capture the instructions regarding recall time of 4 weeks, and the “Don’t apply to me-response options” creates missing without any context to as why they are missing. I suggest these issues are included in both stage 3 and 4 and discussed further. **Thank you for this useful comment. Previous research by Eton et al has shown that adding in a ‘does not apply’ option can reduce amount of missing data in preference to the inclusion of screening questions that allow the participant to skip questions.** **^1^** **The wide-ranging nature of treatment burden unfortunately means that not all burdens are applicable to everyone. We appreciate the challenges that missing data can present.** **We have added the following underlined text to stage 3: Percentage of missing items and proportion of returned surveys will provide information on acceptability, and a subset of 30 participants will be interviewed by telephone after completion of the questionnaire to further explore this (they will tick a box on the form to opt in). Participant responses and missing data will be analysed to explore any possible issues with response options or recall period.** **We have added the following to stage 4: Examples of topics that will be discussed include time taken to complete the survey, wording of instructions and items, mode of survey (paper and pencil), whether any breaks or help from a proxy were required, and any issues with response options or recall period.**

 5. Figures: The figures are informative. Even though the methods section is written in a clear manner, I would welcome a figure giving an overview of the study with it’s 4 stages including procedures and participants would be helpful to the readers functioning as a visual model of the 4 stages. **Thank you, we have amended Figure 1 to show how the 4 stages described in the manuscript correlate to the Figure. **  

 1. Eton DT, Lee MK, St Sauver JL, Anderson RT. Known-groups validity and responsiveness to change of the patient experience with treatment and self-management (pets vs. 2.0): A patient-reported measure of treatment burden. *Qual Life Res*. 2020;29:3143-3154

# Reviewer report: Reviewer response for version 1

**DOI:** 10.21956/healthopenres.14418.r27378

**Published:** 2023-07-13

**Authors:** Adrià Arboix

**Affiliations:** 1 Universitat de Barcelona, Barcelona, Spain

The authors present a study aimed to adapt, refine, and validate the Patient Experience with Treatment and Self-Management (PETS) measure to develop a new patient-reported measure (PRM) of treatment burden for use with stroke survivors. The authors concluded that PETS-stroke is designed for use as an outcome measure in clinical trials of stroke treatments and complex interventions to ascertain if treatments are feasible for patients in the context of their daily lives. The study is potentially interesting, but can be improved if the following considerations are addressed:

1. The authors should indicate that an essential line of future research would be precisely the assessment of the role of PETS-stroke among adult stroke patients with ischemic lacunar versus non-lacunar stroke. This recommendation is because the pathophysiology, prognosis and clinical features of lacunar strokes are different from other acute cerebrovascular diseases (Rudilosso *et al.*, 2022 ^1^). Did the authors consider this in their study protocol?
2. It would be interesting if the authors included in the text some of the limitations of this study.  
3. A brief concluding comment on other possible lines of future research on the presented topic would be appreciated.

Is the study design appropriate for the research question?

Yes

Is the rationale for, and objectives of, the study clearly described?

Yes

Are sufficient details of the methods provided to allow replication by others?

Yes

Are the datasets clearly presented in a useable and accessible format?

Yes

Reviewer Expertise:

cerebrovascular diseases; acute stroke; vascular cognitive impairment; lacunar stroke

I confirm that I have read this submission and believe that I have an appropriate level of expertise to confirm that it is of an acceptable scientific standard.

## References

[1] : The Potential Impact of Neuroimaging and Translational Research on the Clinical Management of Lacunar Stroke. *Int J Mol Sci* .2022;23(3) : 10.3390/ijms23031497 10.3390/ijms23031497 35163423 PMC8835925

# Response

**Published:** 2023-08-22

**Authors:** Martin Taylor-Rowan

**Affiliations:** General Practice and Primary Care, University of Glasgow, Glasgoe, UK

1. The authors should indicate that an essential line of future research would be precisely the assessment of the role of PETS-stroke among adult stroke patients with ischemic lacunar versus non-lacunar stroke. This recommendation is because the pathophysiology, prognosis and clinical features of lacunar strokes are different from other acute cerebrovascular diseases (Rudilosso et al., 20221). Did the authors consider this in their study protocol?
**Thank you, we have added the following to the section ‘outputs and future work’: It would also allow examination of sociodemographic risk factors for treatment burden such as socioeconomic deprivation or ethnicity, and comparison of treatment burden in different stroke types, for example ischaemic versus haemorrhagic or lacunar versus non-lacunar stroke.**  
2. It would be interesting if the authors included in the text some of the limitations of this study.   
**Thank you, as this is a protocol paper, we felt a section on ‘potential challenges’ would be suitable. We have added the following *: *One possible challenge is reaching our recruitment target and to mitigate this we are using a multifaced approach to recruitment. Another challenge is missing data, which will be minimized through careful content validation and any missing data will be scrutinized for possible causes. Lastly, representation of the sample will be closely examined.
**  
3. A brief concluding comment on other possible lines of future research on the presented topic would be appreciated.  
**Thank you, we have added to the section ‘outputs and future work’ as stated above. **
